# The Anti-Tumorigenic Activity of Sema3C in the Chick Embryo Chorioallantoic Membrane Model

**DOI:** 10.3390/ijms20225672

**Published:** 2019-11-12

**Authors:** Indrė Valiulytė, Rūta Curkūnavičiūtė, Laura Ribokaitė, Arunas Kazlauskas, Monika Vaitkevičiūtė, Kęstutis Skauminas, Angelija Valančiūtė

**Affiliations:** 1 Neuroscience Institute, Medical Academy, Lithuanian University of Health Sciences, LT-50161 Kaunas, Lithuania; arunas.kazlauskas@lsmuni.lt (A.K.); mvaitkev@gmail.com (M.V.); kestutis.skauminas@lsmuni.lt (K.S.); 2 Department of Histology and Embryology, Lithuanian University of Health Sciences, LT-44307 Kaunas, Lithuania; ruta.curkunaviciute@lsmu.lt (R.C.); laura.ribokaite@fc.lsmuni.lt (L.R.); angelija.valanciute@lsmuni.lt (A.V.)

**Keywords:** Sema3C, sodium valproate, chicken embryo chorioallantoic membrane model, glioblastoma, angiogenesis, invasiveness, cell proliferation, cell adhesion

## Abstract

Sema3C protein, a member of the class 3 family of secreted semaphorins, play an important role in tumor development by regulating cell proliferation, migration, invasion, and angiogenesis processes. Depending on the type and malignancy grade of the tumor, Sema3C function remains controversial. In this study, we constructed a stably overexpressing Sema3C glioblastoma cell line U87 MG and tested it on the chicken embryo chorioallantoic membrane (CAM) model with the aim to reveal Sema3C protein function on angiogenesis process in ovo. Our experiments showed that Sema3C not only affects angiogenesis of CAM by inhibiting neovascularization but also acts as an anti-tumorigenic molecule by hampering U87 MG cell invasion into mesenchyme. The effects of Sema3C on CAM were similar to the effects of anti-epileptic drug sodium valproate (NaVP). Both, anti-angiogenic and anti-tumorigenic activities of Sema3C were enhanced by the treatment of NaVP and, importantly, were not attributed to the cytotoxic effects. Our studies suggest that Sema3C could be a promising target for glioblastoma treatment.

## 1. Introduction

Class 3 semaphorin proteins (Sema3) belong to the family of secreted transmembrane glycoproteins. They consist of seven members (A–G) which are found in most tissues, including cardiovascular, endocrine, gastrointestinal, liver, skeletal, kidney, reproductive, and respiratory systems but mostly in the nervous system, especially during its development [1,2]. Sema3 proteins are essential for the development and normal functioning of organs and play an important role in pathologies, such as Alzheimer’s disease, schizophrenia, Parkinson’s disease, various cancer types, and may also evoke epilepsy, neuronal degeneration, vascular and cardiac diseases [1]. In tumor biology, it was shown that Sema3 family members can regulate tumor cell proliferation, migration, survival, metastasis, lymphangiogenesis, and angiogenesis processes in different ways [3,4]. The different actions of individual semaphorins depend on the type of the tissue, malignancy of the tumor, and the proteolytical cleavage of the Sema3 molecules [5]. One member, Sema3C protein, has been shown to be involved in the development of gastric, breast, pancreatic, brain, prostate, and other cancers [6], as well as in maintenance of cancer stem-like cells (CSCs) [7]. Furthermore, Sema3C protein inhibits pathological retinal angiogenesis through the Neuropilin-1 and PlexinD1 receptors [8] and inhibits lymphangiogenesis by inducing the collapse of the cytoskeleton of lymphatic endothelial cells by the Neuropilin-2, PlexinA1, and PlexinD1 receptors [9]. Full length Sema3C mutant resistant to cleavage by furin, inhibits choroidal neovascularization in age-related macular degeneration [10]. Recently, we also revealed that Sema3C protein inhibits microcapillary formation in an in vitro system and the C-terminal arginine of the putative furin cleavage site _742_RNRR_745_ at the basic domain of Sema3C protein is critical for its functions in angiogenesis process [11]. However, the function of Sema3C protein in tumorigenesis remains controversial. For example, it was demonstrated that higher levels of Sema3C are associated with the progression of astrocytoma [12] and gastric cancer, the latter probably is due to the stimulation of angiogenesis processes [13]. Moreover, Sema3C promotes survival and invasion of glioma stem cells (GSCs) through Rac1 activation [14].

Glioblastoma (GBM) is a highly vascularized tumor with poor survival prognosis [15]. Results of our previous experimental study [16] using a chicken embryo chorioallantoic membrane (CAM) model have demonstrated that U87 MG cells elevate angiogenesis processes and this effect was inhibited with therapeutic agent sodium valproate (NaVP) which interferes with the p53 and EZH2 molecular pathways important for glioma progression. NaVP has a function in decreasing the U87 MG cell proliferation, migration, and angiogenesis, thereby holding promise of NaVP potential in glioblastoma therapy. However, NaVP is a synthetic drug, with a low improvement in overall survival rate (approximately 2.4 month) [17], making it necessary to find more potent anti-angiogenic molecules for cancer treatment. Therefore, in this study we wanted to reveal the functional role of Sema3C protein in gliomagenesis and angiogenesis processes. To achieve this goal, on the basis of glioblastoma U87 MG cells we constructed Sema3C-overexpressing stable cell line U87t-Sema3C, which we tested in the CAM model.

## 2. Results

### 2.1. Construction and Verification of the U87t-Sema3C Cell Line

**Figure 1:**
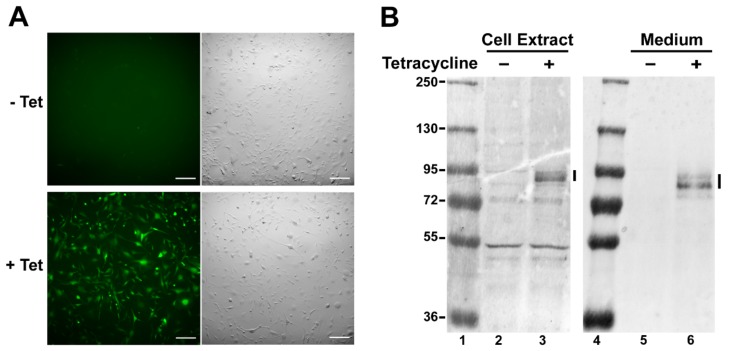
Verification of the constructed U87t-Sema3C cell line. (**A**) Microscopic images. Pictures were taken after 2 days of incubation with tetracycline. Fluorescence indicates overexpression of Sema3C protein. -Tet—no tetracycline, +Tet—1 µg/mL of tetracycline was added to the cell media. Scale bar, 100 μm. (**B**) Western blot analysis of Sema3C protein expression in U87t-Sema3C cells and cell medium. Black bar indicates Sema3C protein, approximately 85 kDa in size. Lanes 1 and 4—protein marker; lanes 2 and 5—untreated cells; lanes 3 and 6—cells treated with 1 µg/mL of tetracycline.

To evaluate the role of Sema3C in gliomagenesis and angiogenesis processes, we generated a stable U87t-Sema3C cell line on the basis of the glioblastoma U87 MG cells, which express Sema3C and yellow fluorescing protein Venus in a tetracycline-inducible manner (Section 4). After 48 h of incubation with tetracycline, U87t-Sema3C cells exhibited fluorescence emitted by newly synthesized Venus protein (Figure 1A). In order to verify that the U87t-Sema3C cell line synthesizes the Sema3C protein in addition to Venus, the Western blot analysis were performed on tetracycline-treated and untreated (control) cells. With Sema3C-specific antibodies we detected the signal corresponding to the predicted size (approximately 85.2 kDa) of Sema3C protein in the tetracycline-induced cell extract, whereas the signal was not presented in the non-induced cell sample (comparing lanes 2 and 3 in Figure 1B). Since Sema3C is a secreted protein, we used the same antibodies to check if it is transported to the extracellular milieu. The results showed that the protein signal was detected only in the sample of media collected from tetracycline-induced cells (comparing lanes 5 and 6 in Figure 1B). Taken together, the results of fluorescence microscopy and Western blot analysis proved that we successfully generated the stable U87t-Sema3C cell line, which synthesizes Sema3C protein in a tetracycline-induced manner and this protein is successfully transported to the extracellular media.

### 2.2. Sema3C and NaVP Effects on Vascularization and Invasiveness of U87 Cell-Formed Tumors

**Figure 2:**
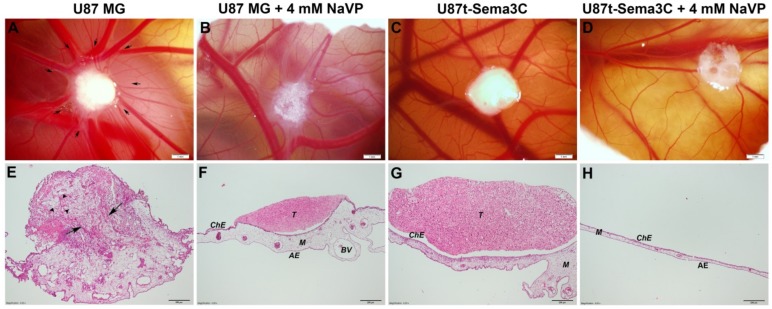
Biomicroscopy in vivo and microscopic images of the tumors on the chicken embryo chorioallantoic membrane (CAM). (**A**–**D**) pictures show tumor xenografts photographed on day 10 of embryo development via window in eggshell. (**A**) CAM with U87 MG cell-formed tumor (*n* = 17). Tumor has attracted blood vessels of the CAM, new vessels have developed, and the “spoked-wheel” pattern of blood vessel distribution to the direction of the tumor is clearly visible. Blood vessels are pointed with arrows. (**B**) CAM with U87 MG cell tumor treated with 4 mM sodium valproate (NaVP) (*n* = 10). “Spoked-wheel” pattern of blood vessels has diminished; tumor was located on the surface of the CAM. (**C**) CAM with U87t-Sema3C cell-generated tumor (*n* = 11). (**D**) CAM with U87t-Sema3C cell tumor treated with 4 mM NaVP (*n* = 13). The “spoked-wheel” pattern of blood vessel distribution is not visible in pictures (**C**) and (**D**–**H**) hematoxylin and eosin staining show tumor invasion into the CAM and adhesion to the CAM surface at day 12 of embryo development. (**E**) CAM with U87 MG tumor that completely invaded CAM mesenchyme. Arrowheads point to the chicken blood vessels formed in the tumor, and long arrows show the destruction of the integrity of chorionic epithelium by tumor cells. (**F**) CAM with U87 MG cell tumor treated with 4 mM NaVP. (**G**) CAM with U87t-Sema3C tumor. In (**F**) and (**G**) pictures, non-invaded and not vascularized tumor is shown on top of the CAM and the integrity of chorionic epithelium is intact. (**H**) Tumor generated by U87t-Sema3C cells failed to adhere to CAM surface upon 4 mM NaVP treatment. Not adhered tumor separated at EDD12 during CAM collection for histology. ChE: Chorionic epithelium, AE: Allantoic epithelium, BV: Blood vessels, M: Mesenchyme, and T: Tumor. Scale bars: (**A**–**D**)—1 mm; (**E**–**H**)—200 μm. The white material visible in microscopy panels are remains of the surgical sponge material.

In line with our previous findings, U87 MG cells formed tumors on the CAM [12], which attracted mesenchymal blood vessels forming a well-defined “spoked wheel” pattern of distribution to the direction of the tumor (Figure 2A). Borders of the tumor are not clear because it has invaded the CAM mesenchyme. Blood vessels penetrated the tumor and could be observed in the hematoxylin- and eosin-stained histological image (Figure 2E). U87 MG cell tumors treated with 4 mM of NaVP had a well-marked shape with clear borders and also attracted mesenchymal blood vessels but “spoked-wheel” pattern diminishes. Importantly, the tumors were not vascularized (Figure 2B,F). U87t-Sema3C cell-produced tumors did not induce new blood vessel formation on CAM in both non-treated and treated with 4 mM of NaVP groups (Figure 2C,D, respectively). CAM mesenchyme in these samples was vascularized, but blood vessels did not have orientation towards the tumor (Figure 2G,H, respectively).

**Figure 3:**
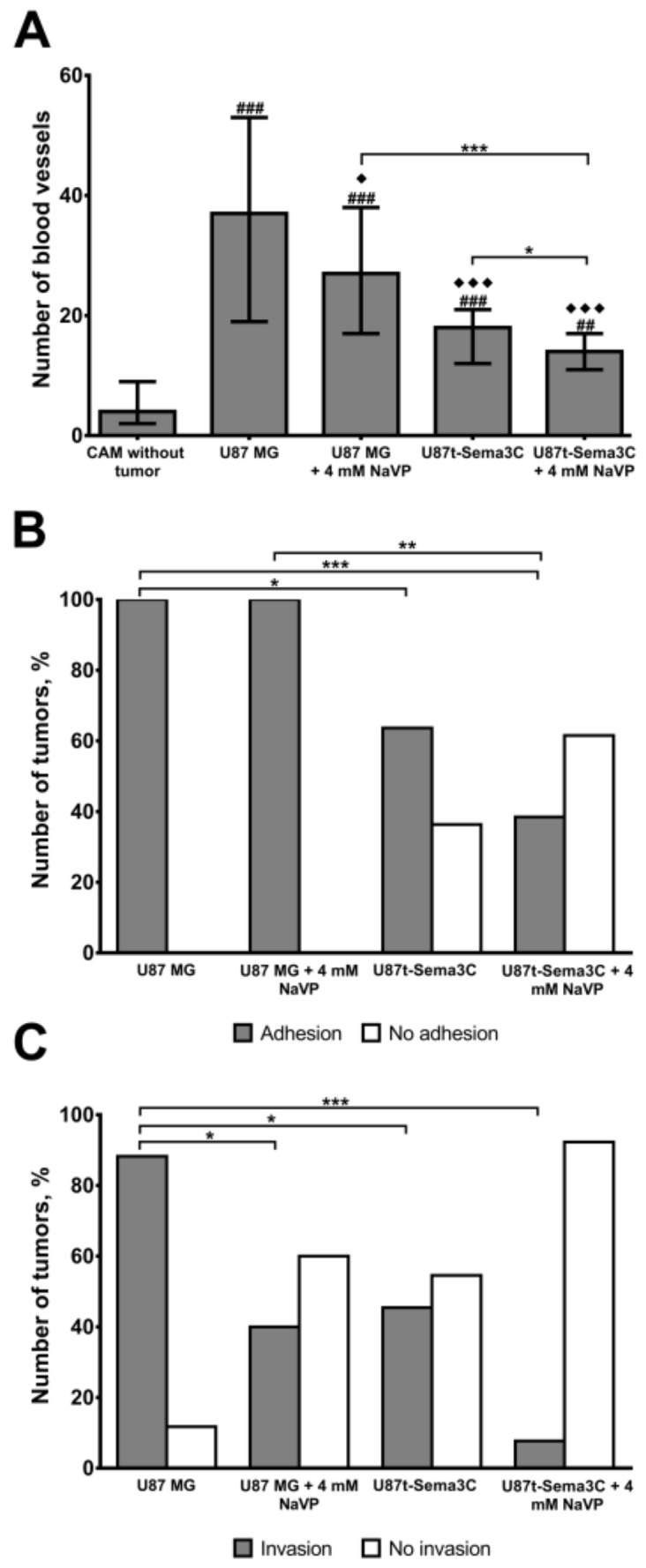
Effects of Sema3C and NaVP to the frequency of U87 MG cell tumor blood vessel formation in the mesenchyme of CAM (**A**), adhesion to CAM (**B**), and invasion into CAM (**C**). The investigated groups were: CAM without tumor (*n* = 10), U87 MG cell tumors (*n* = 17), U87 MG cell tumors treated with 4 mM of NaVP (*n* = 10), U87t-Sema3C cell tumors (in picture (**A**), *n* = 7; in picture (**B**) and (**C**), *n* = 11), and U87t-Sema3C tumors treated with 4 mM of NaVP (in picture (**A**), *n* = 5; in picture (**B**) and (**C**), *n* = 13). The data in picture (**A**) are presented as medians with a range; #—compared to the group of CAMs without tumor, ^##^ *p* < 0.01, ^###^ *p* < 0.001; ◆—compared to the control group of U87 MG cells, ^◆^ *p* < 0.05, ^◆◆◆^ *p* < 0.001; * *p* < 0.05, ** *p* < 0.01, *** *p* < 0.001.

Figure 3A shows vascularization of the CAM mesenchyme estimated in histological samples of the Figure 2 experiment. The cases when tumors did not adhere to the CAM where excluded. Results showed that U87 MG cells significantly increased the formation of blood vessels in the mesenchyme of the CAM, compared to the CAM without tumor (the median of blood vessels 37 with a range of 19–53, *p* < 0.001). The vascularization was reduced upon treatment of 4 mM of NaVP (median 27 with a range of 17–38, *p* < 0.05) and/or in the presence of Sema3C protein (median 18 with a range of 12–21, *p* < 0.001). Importantly, the synergistic effects of Sema3C and NaVP were observed on vascularization (*p* < 0.05), where the median of blood vessels was the lowest, compared to U87 MG group (median 14 with a range of 11–17, *p* < 0.001). Figure 3B,C shows estimated effects of Sema3C and NaVP on the frequency of U87 MG cell tumor adhesion to and invasion into the CAM, respectively. All non-treated and NaVP-treated U87 MG tumors adhered firmly to the CAM epithelium (Figure 3B). U87t-Sema3C cell-formed tumors adhered to the CAM epithelium in 63.64% of cases (*p* < 0.05) and upon NaVP treatment U87t-Sema3C tumors adhered to the CAM epithelium only in 38.46% of cases (*p* < 0.001, Figure 3B). In most cases (85.71%), control U87 MG cell tumors invaded CAM mesenchyme or destroyed chorionic epithelium (Figure 3C). Under the influence of 4 mM of NaVP, invasion was diminished down to 40% of cases (*p* < 0.05). Non-treated U87t-Sema3C tumors invaded CAM in 45.45% of cases (*p* < 0.05), whereas upon the treatment with 4 mM of NaVP, tumor invasion was observed only in 7.69% of cases (*p* < 0.001, Figure 3C). Tumor adhesion to the CAM epithelium without invasion is shown in Figure 2F,G.

### 2.3. Effects of Sema3C and NaVP on Glioblastoma U87 Cell Invasion In Vitro

**Figure 4:**
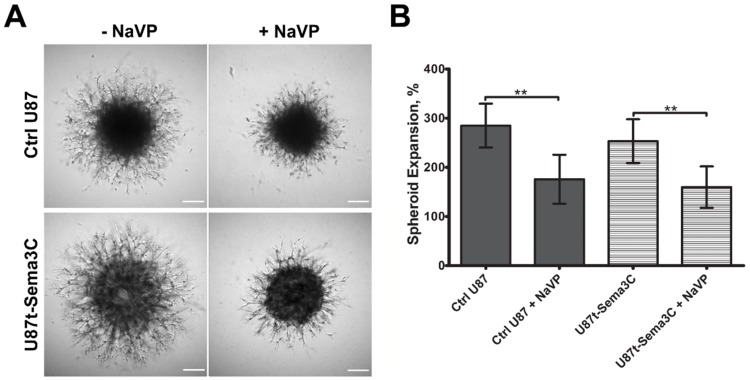
Analysis of U87 and U87t-Sem3C cells invasion into Geltrex. The inhibition of glioblastoma (GBM) cell invasion by NaVP was detected in the absence or presence of Sema3C overexpression. (**A**) Representative bright field microscopy images of control U87 (Ctrl U87) and U87t-Sem3C cell spheroids under different treatments, as indicated. Scale bar, 100 μm. (**B**) Graphic representation of the spread of spheroidal cells indicated as Spheroid Expansion. N = 6 in each treatment group. Data are presented as means ± SD; ** *p* < 0.01.

We wanted to verify effects of Sema3C and NaVP on GBM cell invasion process by using an in vitro system. For this purpose, we generated U87t and U87t-Sema3C cell spheroids, covered them with the extracellular matrix-mimicking gel Geltrex, the major components of which include laminin, collagen IV, entactin, and heparin sulfate proteoglycans and incubated for five days with DMEM medium, supplemented with 0.1 µg/mL of tetracycline and with or without 4 mM of NaVP. As shown in Figure 4A, control U87 and U87t-Sema3C cells sprouted and spread widely with the similar extent around the original shape of the spheroid. NaVP significantly inhibited both control U87 and U87t-Sema3C cell invasion into the Geltrex to a similar extent (*p* < 0.01; Figure 4A,B).

### 2.4. Effects of NaVP on U87t-Sema3C Cell Viability and Proliferation

**Figure 5:**
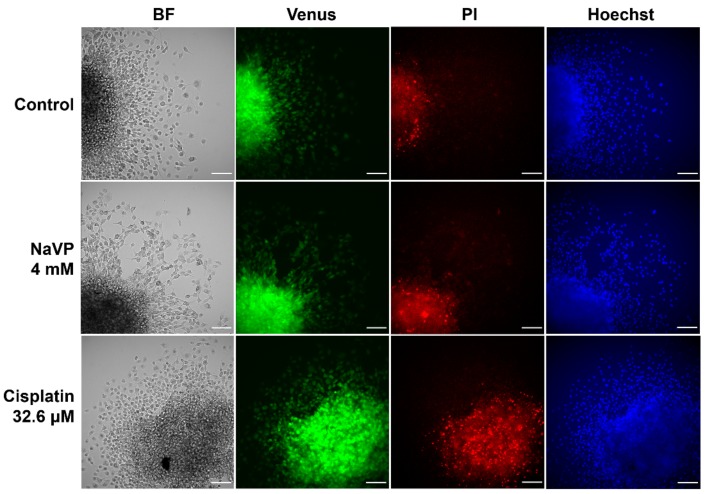
U87t-Sem3C cell viability. Green fluorescence indicates live, Sema3C protein overexpressing cells; propidium iodide (PI) indicates bright red necrotic cells; blue fluorescence indicates cell nucleus stained with Hoechst. BF—bright-field; Scale bar, 100 μm.

As we found previously that Sema3C has a tumor-inhibiting activity by suppressing tumor-associated angiogenesis processes on CAM model and this effect was even stronger with NaVP, we wanted to evaluate the cytotoxicity of NaVP on glioblastoma cells. For this purpose, U87t-Sema3C cell spheroids were placed onto Petri dishes and allowed to spread for two days of incubation and were subsequently stained with fluorescent Hoechst and propidium iodide dyes. A total of 4 mM of NaVP was used to reveal the effect of NaVP on cell viability. As a positive cell toxicity control, we used cisplatin (32.6 µM), since this drug is well known as chemotherapeutic agent used for cancer treatment by inducing apoptosis [18]. As shown in Figure 5, control U87t-Sema3C cells spread widely and were fluorescing green indicating that cells were overexpressing Sema3C protein. Moreover, the propidium iodide assay demonstrated almost none of necrotic cells (no bright red color) and Hoechst staining showed homogenous blue color of nucleus of live cells. In the presence of 4 mM of NaVP, U87t-Sema3C cells were morphologically similar to control cells. Propidium iodide staining showed only a few necrotic cells, suggesting that NaVP at 4 mM concentration as well as Sema3C expression did not alter the morphological changes of GBM cells and are not associated with cell cytotoxicity. As we expected, 32.6 µM of cisplatin caused U87t-Sema3C cell morphological changes. Cells became round-shaped and propidium iodide staining showed a high number of necrotic cells.

**Figure 6:**
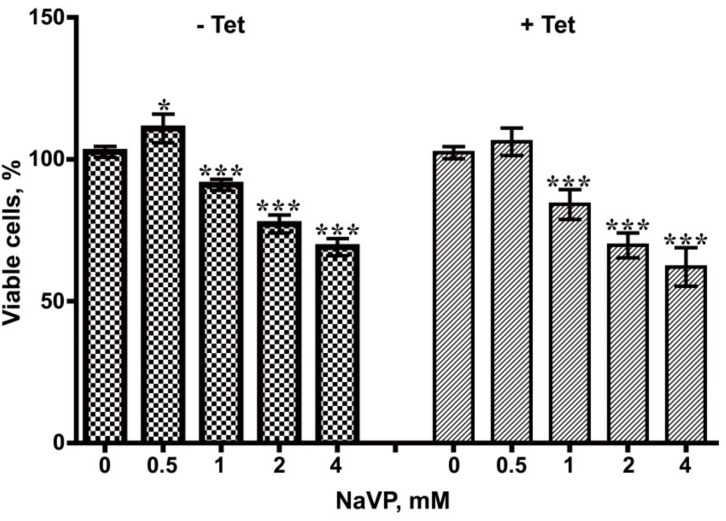
U87t-Sem3C cell viability/proliferation test. Number of viable cells decreased with increased amount of NaVP. Tet—tetracycline. Data are presented as means ± SD; * *p* < 0.05; *** *p* < 0.001, compared to 0 point.

To asses if NaVP together has an effect on glioblastoma cell viability/proliferation in the presence and absence of Sema3C expression, tetracycline-induced and non-induced U87t-Sema3C cells were treated with different concentrations of NaVP (0–4 mM) over 72 h and the MTT assay was carried out. As shown in Figure 6, NaVP inhibited growth of GBM cells in both cases in a dose-dependent manner. The most pronounced inhibition of non-induced and tetracycline-induced U87t-Sema3C cell viability was obtained with 4 mM concentration of NaVP (30.95% and 37.90%, respectively; *p* < 0.001, respectively). Interestingly, Sema3C protein had no statistically significant influence on the NaVP effect.

## 3. Discussion

Malignant tumors, including glioblastomas, tend to be highly vascularized [15]. Therefore, there is a need to find tumor-specific molecules that could be molecular therapeutic targets for anti-angiogenic therapy. Sema3 play an important role in cancer biology by interacting with neuropilins, plexins receptors, and VEGF signaling pathway components and regulating cell proliferation, migration, invasiveness, and angiogenesis processes [3]. Depending on the specificity of the tissue, malignancy of the tumor, and proteolytic cleavage of individual Sema3 molecules, diverse Sema3 proteins could have opposing roles in cancer development [5,6]. Our previous experiment showed that one of the members of the class 3 family of semaphorins, Sema3C, significantly inhibited microcapillary structure formation in an in vitro assay [11]. Other research groups also noticed anti-angiogenic effects of Sema3C protein on pathological blood vessel formation of mouse retina [8], in lymphangiogenesis [9], and choroidal neovascularization processes [10]. According to Yang et al., Sema3C inhibits endothelial cell junction integrity, migration, and focal adhesion with the extracellular matrix through the FAK and p38MAPK signaling pathways [8]. In this study, we aimed to elucidate role of Sema3C in glioma associated angiogenesis process by applying the Sema3C-overexpressing derivative of the glioblastoma cell line U87 MG on a CAM model. Glioblastoma cell line U87 MG was recently shown by our research to effectively form invasive tumors and promote angiogenesis of CAM, and these effects were inhibited by NaVP [16]. In the current study, Sema3C overexpression by U87 MG cells produced inhibitory effect of blood vessel formation, tumor invasion, and adhesion in ovo similar to the effects elicited by NaVP (Figure 2 and Figure 3). Moreover, anti-tumorigenic and anti-angiogenic effects of NaVP in ovo were enhanced in the presence of Sema3C overexpression, where the tumor cells placed on CAM failed to adhere to its surface (Figure 2H). In vitro experiments confirmed the inhibitory effect of NaVP on U87 cell invasion, which, noteworthy, was not enhanced by the Sema3C overexpression as it was observed in in ovo experiments (Figure 4). This suggests that the certain cellular environment is required for Sema3C to augment inhibitory effects on tumor cell migration.

NaVP has been widely recognized as a therapeutic agent for the treatment of epilepsy, bipolar disorder, neuralgia, and migraine [19,20]. Other studies have shown NaVP to exert anticancer and antiangiogenetic properties [21]. It also has been found that NaVP inhibits histone deacetylases (HDACs), enzymes that are responsible for adding or removing acetyl groups to or from lysine residues of target histones and non-histone proteins [22,23]. Such an epigenetic modification is a novel therapeutic target in glioblastoma [24]. Inhibition of HDAC has been shown to induce GBM tumor cell apoptosis, suppress cell growth, and sensitize cells to radiotherapy and chemotherapy. It is done by an increasing production of reactive oxygen species, p21 expression, G2/M cell cycle arrest, suppressing the repair of DNA double strand break, and activating pro-apoptotic signaling [25,26,27,28]. It was shown that NaVP alone and in combination with other chemotherapeutic drugs has an antiangiogenetic and antiproliferative effect on GBM cells and on in vivo rodent glioma tumors [29,30,31]. In line with these studies, we observed an antiproliferative effect on GBM cells by NaVP treatment (Figure 6), without, however, significant effects on cell viability under our experimental conditions (Figure 5). Importantly, U87 MG cells placed onto CAM seem to undergo dramatic morphological changes upon the treatment with NaVP (Figure S1). We hypothesize that in GBM tumors, both Sema3C overexpression and NaVP treatment operate via the same epithelial-to-mesenchymal transition mechanism as was shown in other studies with prostate cancer [32,33]. When in combination, Sema3C and NaVP components seem to have a synergistic effect on tumor development. However, more experiments on molecular mechanism are needed to confirm this hypothesis.

The biological function of Sema3C in the process of gliomagenesis is yet to be elucidated. There are some studies in which Sema3C is associated with GBM tumor progression. For example, Vaitkiene et al. found an increase of Sema3C protein level in GBM tumor specimens, which is associated with the poor patient survival prognosis [12]. However, it is not clear if the high amount of Sema3C protein represents a functionally active form since the degraded products were detected in immunoblots in glioma specimens. Importantly, Sema3C mRNA expression was not increased in GBM samples, compared to lower grade gliomas. Similar results were shown in other study, where Sema3C mRNA expression was similar between lower-grade and higher-grade gliomas [34]. These discrepancies between mRNA and protein expression levels could be partially explained by the general mechanism of anticancer factor suppression by tumor cells. Nevertheless, other studies suggest that Sema3C’s functional role is dose-dependent and the expression level of Sema3C could affect the ability to connect different effectors and activate diverse signaling pathways. For example, higher Sema3C levels in castration-resistant prostate cancer cells as well as in glioblastoma U87, bladder T24, and kidney Caki-2 cells activated phosphorylation of MET and EGFR, in a dose-dependent manner. Moreover, higher levels of Sema3C protein induced prostate tumor growth and inhibited apoptosis by transactivating multiple receptor tyrosine kinases via PlexinB1 and NRP1 interaction [35], as well as increased progression of pancreatic cancer through extracellular signal-related kinase (ERK) signaling [36]. In GSC, higher doses of recombinant human Sema3C protein increased cell viability and ability to form tumorspheres [14], maintained prostate CSCs [32]. In conclusion, our results revealed that overexpression of Sema3C significantly inhibited GBM-associated tumorigenesis and angiogenesis processes in a CAM model and enhanced the inhibitory effect of therapeutic agent NaVP on GBM cell invasion and adhesion processes. This suggests that Sema3C protein could be a promising target for GBM treatment.

## 4. Materials and Methods

### 4.1. Cell Lines and Chemicals

Glioblastoma U87 MG cells obtained from the European Collection of Cell Cultures (ECACC, cat. No. 89081402) were used to generate Sema3C stably expressing U87t-Sema3C cell line. Cells were cultivated in Dulbecco’s Modified Eagle’s Medium (DMEM; Gibco, Carlsbad, CA, USA), supplemented with 10% fetal bovine serum (FBS; Gibco), 100 IU/mL of penicillin, and 100 µg/mL of streptomycin (p/s; Gibco). Cells were incubated at 37 °C in a humidified atmosphere of 95% air and 5% CO_2_. A total of 1 µg/mL of tetracycline was added to the cell media and incubated for 48 hours in order to make U87t-Sema3C cells to overexpress Sema3C protein.

### 4.2. Generation of Tetracycline-Inducible U87t-Sema3C Cell Line

The generation of a stable U87t-Sema3C cell line, encoding a tetracycline-regulated Sema3C expression system, was based on the T-Rex System by Invitrogen (Invitrogen, Carlsbad, CA, USA, cat. No. K1020-01) and was carried out in two steps. Firstly, U87 MG cells were transfected with the vector pcDNA6/TR encoding the Tet repressor (TetR) and blasticidin resistance gene by using Lipofectamine 2000 Reagent (Invitrogen, Carlsbad, CA, USA). After 48 hours of transfection, cells were incubated with DMEM media supplemented with 6 μg/mL of blasticidin (Gibco) for two weeks to select stable cells. The resultant cell line, termed U87t, provided high-level expression of the TetR proteins that bind to Tet operator 2 (TetO_2_) and repress the transcription of the test-gene Xpress-β-gal (Figure S2). In the presence of tetracycline, TetR homodimers change their conformation, release from TetO_2_, and induce the transcription of the gene of interest. In the second step, the generation of Sema3-expressing cell line U87t-Sema3C was carried out by transfecting the U87t cell line with the bicistronic vector pTO/Sema3C-IRES2-Venus [11], which encodes Sema3C and yellow fluorescing protein Venus separated by the IRES2 element and zeocin resistance gene. After 48 hours of transfection, cells were incubated with DMEM media supplemented with 500 μg/mL of zeocin (Gibco) to select stable cells. After two days, cells were seeded into the 55 cm^2^ Petri plate, and because of the zeocin resistance gene, cells were derived by cultivation with DMEM medium, supplemented with 500 μg/mL of zeocin (Gibco) for two weeks to select stable cells. At the end of the cell selection, cells formed colonies, which were propagated and tested for the tetracycline-inducible expression of Venus under fluorescence microscope (Lumascope LS620, Etaluma, Carlsbad, CA, USA) and Sema3C protein by Western blot analysis.

### 4.3. Western Blot Analysis

The overexpression of Sema3C protein in U87t-Sema3C cells and secretion to the cell medium was analyzed by Western blot. Before analysis, U87t-Sema3C cells were seeded onto 22 cm^2^ Petri dishes and incubated with 1 µg/mL of tetracycline or without it (control) for 48 hours. Cells were scratched off the plates in ice-cold phosphate buffered saline (PBS), suspended in RIPA lysis buffer (50 mM TrisHCl (pH 7.5), 150 mM NaCl, 1% Igepal CA-630, 0.5% sodium deoxycholate, 0.1% SDS), supplemented with Halt Protease Inhibitor Cocktail (ThermoFisher Scientific, Rockford, IL, USA, cat. No. 87785), and centrifuged for 40 min at 12.000 g, 4 °C. The concentration of whole cell extract (WCE) proteins (supernatant) was measured by using Bradford reagent (SERVA, Heidelberg, Germany). Finally, 80 µg of WCE protein were denatured with Laemmli buffer (4% SDS, 20% glycerol, 10% 2-mercaptoethanol, 0.004% bromophenol blue and 0.125 M Tris HCl, pH 6.8) and stored at –80 °C. The media collected from Sema3C-containing and control U87t-Sema3C cells were concentrated to 20-fold with Pierce protein concentrators PES 10K MWCO (Thermo-Fisher Scientific, cat. No. 88513), according to the manufacturer’s protocol. A total of 20 µl of concentrated cell media samples were denatured with Laemmli buffer and stored at –80 °C. Denatured protein samples were loaded onto 7.5% SDS-PAGE and transferred to a nitrocellulose membrane. The membrane was blocked with 10% non-fat milk in PBS overnight followed by incubation with Sema3C rabbit polyclonal antibody (ThermoFisher Scientific, cat. No. PA5-24997, dilution 1:500) for 2 h at room temperature. After washing with PBS-T buffer (PBS supplemented with 0.5% Tween-20), the membrane was incubated with secondary HRP-conjugated goat anti-rabbit antibody (Invitrogen, cat. No. 65-6120, dilution 1:2000) for 40 min at room temperature. Protein signals were visualized using TMB substrate (Sigma-Aldrich, St. Louis, MO, USA, cat. No. T0565) and captured with the digital scanner.

### 4.4. In Ovo CAM Model and Study Groups

Fertilized chicken eggs (Cobb 500) were acquired from a local hatchery and placed in an incubator (Maino incubators, Oltrona di San Mamette, Italy) at 37 °C temperature and 60% relative air humidity. An automatic rotator was used to roll the eggs in an incubator once per hour until day 3 of embryo development (EDD3). At EDD3 the automatic rotation was stopped, eggshells were cleansed with prewarmed 70% ethanol, a small hole was drilled in the location of an air chamber, and approximately 2 mL of albumin were withdrawn with a sterile syringe in order to detach the CAM from the shell. Afterwards, a small square of approximately 1 cm^2^ was drilled, the shell of the egg was carefully removed, and a created window was sealed with a sterile cellophane tape. The eggs were placed back into the incubator until further placement of tumor cells onto the CAM at the EDD7. The following 4 study groups were investigated: (1) U87 MG (*n* = 17), (2) treated with 4 mM of NaVP (*n* = 10), (3) U87 Sema3C (*n* = 11), (4) treated with 4 mM of NaVP (*n* = 13).

### 4.5. The Placement of U87 MG and U87t-Sema3C Cells onto the CAM

The number of 1 × 10^6^ cells was resuspended in 10 µL of serum-free DMEM and 10 µL of the type I rat tail collagen (Gibco, Gaithersburg, MD, USA) was added to the cell suspension. An absorbable surgical sponge (Surgispon, Aegis Lifesciences, India) was cut manually with a blade to form pieces of 9 mm^3^ (3 × 3 × 1 mm). A 20 µL liquid mixture of tumor cells was gently pipetted onto a piece of a surgical sponge. Cells were treated with 4 mM NaVP (Sigma-Aldrich) prior tumor formation (tumor cells were mixed with NaVP solution) and NaVP remained during all experiment. At EDD7 formed tumors (one per chicken embryo) were deposited onto the CAM among major blood vessels. After 5 days of incubation at EDD12 the specimens were harvested, fixed in a buffered 10% formalin solution for 24 hours and embedded into the paraffin wax. The specimens were cut into thin sections of 3 µm of thickness with a microtome (Leica Microsystems Inc., Buffalo Grove, IL, USA), stained with hematoxylin and eosin (H–E), and mounted with a mounting media (Roti HistoKit II, Carl Roth GbmH, Karlsruhe, Germany).

### 4.6. Biomicroscopy In Vivo and Histomorphometric Assay

U87 cell tumors grafted on CAMs of chick embryos were observed in ovo daily and simultaneously from day 2 (EDD9) after tumor grafting to day 5 (EDD12) using an Olympus stereomicroscope SZX2-RFA16 (Olympus Corporation), supplied with an Olympus camera DP72 (Olympus Corporation, Tokyo, Japan) for acquiring both still images and video recordings. Examination of histological slides with H–E stained CAMs with tumors was performed using an Olympus light microscope BX40F4 (Olympus Corporation) equipped with an Olympus digital camera XC30 (Olympus Corporation). Recording the daily in ovo visualization of tumor on CAM and capture of H–E stained slides was performed by acquiring images with CellSens Dimension software (version 1.9, Olympus Corporation, Tokyo, Japan). The number of blood vessels in study groups was evaluated by photographing every H–E stained CAM at 4× magnification directly under the grafted tumor. All blood vessels bigger than 10 µm were counted in the CAM of the same length (1792 µm).

### 4.7. Assessment of Tumor Adhesion and Invasion into the CAM Mesenchyme

Tumor adhesion to the surface of chorionic epithelium and invasion into the CAM was assessed in every investigated group. Tumor adhesion was evaluated macroscopically at EDD12 after harvesting the CAM. A tumor was considered as adhered when it was firmly attached to the CAM surface. Tumors freely floating on the surface of the CAM were considered as non-adhered. Invasion was evaluated on H–E stained histological slides. Invasion was recognized as the destruction of chorionic epithelium or/and tumor cell invasion into the CAM mesenchyme. Non-invaded tumors were located on the surface of the CAM, but the integrity of chorionic epithelium was not disrupted.

### 4.8. Cell Viability/Proliferation Assay

The cell sensitivity to NaVP was analyzed by a 3-(4,5-dimethyl-2-thiazolyl)-2,5-diphenyl-2-H-tetrazolium bromide (MTT) assay. Non-induced and tetracycline-induced U87t-Sema3C cells were seeded in 96-well plate (1.5 × 10^3^ cells per well) separately, using DMEM medium. For tetracycline-induced cells, 0.1 µg/mL of tetracycline was additionally added to the medium. After 24 hours, cells were treated with different concentrations of NaVP (0–4 mM) for 3 days. Finally, cells were incubated with 100 µl of MTT (Invitrogen, cat. No. M6494) solution (0.5 mg/mL) for 3 hours and the formazan crystals formed inside cells were dissolved with 100 µl DMSO for 15 min. The optical density was measured at 550 nm and 620 nm with Multiskan GO Microplate Spectrophotometer (ThermoFisher Scientific). Eight replicates were used for each NaVP concentration and the experiment was done three times independently.

### 4.9. Cell Invasion Assay

For cell invasion assay, U87t and U87t-Sema3C cells (5 × 10^3^ cells/well) were seeded in round-bottomed wells of the Nunclon Sphera 96U-well plate (ThermoFisher Scientific, cat. No. 174925) and incubated for 2 days in DMEM medium with or without 1 µg/mL of tetracycline. Completely formed U87t and U87t-Sema3C spheroids were transferred to the flat-bottomed wells of the Nunclon Sphera 96F-well plate (ThermoFisher Scientific, cat. No. 174927) and coated with 50 µl of Geltrex (LDEV-Free Reduced Growth Factor Basement Membrane Matrix, Gibco, cat. No. A1413202). After 30 min incubation, DMEM medium supplemented with or without 0.1 µg/mL of tetracycline and 4 mM of NaVP was filled on top of the Geltrex. Spheroids were incubated for 5 days; the culture media was changed 3 days after seeding. Spheroid pictures were captured with a microscope (Lumascope LS620, Etaluma, Carlsbad, CA, USA) at 10× magnification. 2D cell contour images of spheroids were then generated using Fiji software (https://fiji.sc/; [37]) and areas were measured using the ImageJ software (https://imagej.net/ImageJ, U. S. National Institutes of Health, Bethesda, MD, USA) and spreading of surfaces was evaluated by in 6 spheroids of each treatment group. The percentage of spheroid area expansion comparing the day 1 spheroid to the day 5 spheroid was calculated and the obtained average values from all samples were plotted on the graph (see Figure 4B).

### 4.10. Cell Viability Assay

For cell viability test, U87t-Sema3C cells (5 × 10^3^ cells/well) were seeded in round bottom Nunclon Sphera 96U-well plate and incubated for 2 days in DMEM medium with 1 µg/mL of tetracycline. Completely formed cell spheroids were transferred to regular 6 well plate (3 spheroids/well) with DMEM medium supplemented with 0.1 µg/mL of tetracycline. After 24 hours, cell media was changed to DMEM medium with 4 mM of NaVP or 32.6 µM of cisplatin (Sigma-Aldrich, cat. No. P4394) and 0.1 µg/mL of tetracycline for the next two days. For controls, DMEM medium supplemented with 0.1 µg/mL of tetracycline was used. The morphological changes of U87t-Sema3C cells in spheroids were assessed by propidium iodide (10 μg/mL, PI; Sigma-Aldrich) staining, which shows brightly red colored necrotic cells and Hoechst 33342 (5 μg/mL; Life Technologies, Darmstadt, Germany) staining, which binds to the DNA of live cells (homogenous blue color) and also shows apoptotic cells (bright blue color). Green fluorescence of U87t-Sema3C cells indicates live Sema3C-expressing cells. Results were taken with a fluorescence microscope at 20× magnification.

### 4.11. Statistical Analysis

GraphPad Prism (version 7.0, Graph-Pad Software, Inc., San Diego, CA, USA) and SPSS software (version 25.0, IBM SPSS, Armonk, NY, USA) were used to evaluate the data of the frequency of proliferation, invasion, and adhesion among investigated groups. Results were shown as means ± SD. The chi-squared test was carried out to determine statistically significant data. The normality assumption was verified by the Shapiro–Wilk test. Data are expressed as median and range (minimum and maximum values) because normality assumptions are not met. Differences between two independent groups were evaluated using the nonparametric Mann–Whitney U test. Statistical significance was indicated as *p* < 0.05.

## Supplementary Materials

Supplementary materials can be found at https://www.mdpi.com/1422-0067/20/22/5672/s1.

## Abbreviations

CAM	Chicken Embryo Chorioallantoic Membrane
NaVP	Sodium Valproate
Sema3	Class 3 Semaphorin
GBM	Glioblastoma

## References

[1] Yazdani U., Terman J.R. (2006). The Semaphorins. Genome Biol..

[2] Alto L., Terman J. (2017). Semaphorins and Their Signaling Mechanisms. Methods Mol. Biol..

[3] Neufeld G., Mumblat Y., Smolkin T., Toledano S., Nir-Zvi I., Ziv K., Kessler O. (2016). The Role of the Semaphorins in Cancer. Cell Adh. Migr..

[4] Toledano S., Nir-Zvi I., Engelman R., Kessler O., Neufeld G. (2019). Class-3 Semaphorins and Their Receptors: Potent Multifunctional Modulators of Tumor Progression. Int. J. Mol. Sci..

[5] Nasarre P., Gemmill R.M., Drabkin H.A. (2014). The Emerging Role of Class-3 Semaphorins and Their Neuropilin Receptors in Oncology. Onco. Targets. Ther..

[6] Hui D.H.F., Tam K.J., Jiao I.Z.F., Ong C.J. (2019). Semaphorin 3C as a Therapeutic Target in Prostate and Other Cancers. Int. J. Mol. Sci..

[7] Hao J., Yu J.S. (2018). Semaphorin 3C and Its Receptors in Cancer and Cancer Stem-Like Cells. Biomedicines.

[8] Yang W.-J., Hu J., Uemura A., Tetzlaff F., Augustin H.G., Fischer A. (2015). Semaphorin-3C Signals through Neuropilin-1 and PlexinD1 Receptors to Inhibit Pathological Angiogenesis. EMBO Mol. Med..

[9] Mumblat Y., Kessler O., Ilan N., Neufeld G. (2015). Full-Length Semaphorin-3C Is an Inhibitor of Tumor Lymphangiogenesis and Metastasis. Cancer Res..

[10] Toledano S., Lu H., Palacio A., Ziv K., Kessler O., Schaal S., Neufeld G., Barak Y. (2016). A Sema3C Mutant Resistant to Cleavage by Furin (FR-Sema3C) Inhibits Choroidal Neovascularization. PLoS ONE.

[11] Valiulyte I., Preitakaite V., Tamasauskas A., Kazlauskas A. (2018). Importance of the Putative Furin Recognition Site 742RNRR745 for Antiangiogenic Sema3C Activity in Vitro. Brazilian J. Med. Biol. Res..

[12] Vaitkienė P., Skiriutė D., Steponaitis G., Skauminas K., Tamašauskas A. (2015). High Level of Sema3C Is Associated with Glioma Malignancy. Diagn. Pathol..

[13] Miyato H., Tsuno N.H., Kitayama J. (2012). Semaphorin 3C Is Involved in the Progression of Gastric Cancer. Cancer Sci..

[14] Man J., Shoemake J., Zhou W., Fang X., Wu Q., Rizzo A., Prayson R., Bao S., Rich J.N., Yu J.S. (2014). Sema3C Promotes the Survival and Tumorigenicity of Glioma Stem Cells through Rac1 Activation. Cell Rep..

[15] Takano S., Yamashita T., Ohneda O. (2010). Molecular Therapeutic Targets for Glioma Angiogenesis. J. Oncol..

[16] Kavaliauskaitė D., Stakišaitis D., Martinkutė J., Šlekienė L., Kazlauskas A., Balnytė I., Lesauskaitė V., Valančiūtė A. (2017). The Effect of Sodium Valproate on the Glioblastoma U87 Cell Line Tumor Development on the Chicken Embryo Chorioallantoic Membrane and on EZH2 and P53 Expression. Biomed. Res. Int..

[17] Lu V.M., Texakalidis P., McDonald K.L., Mekary R.A., Smith T.R. (2018). The Survival Effect of Valproic Acid in Glioblastoma and Its Current Trend: A Systematic Review and Meta-Analysis. Clin. Neurol. Neurosurg..

[18] Shaloam D., Tchounwou P.B. (2014). Cisplatin in Cancer Therapy: Molecular Mechanisms of Action. Eur. J. Pharmacol..

[19] Mba G.M.P., Naunton M. (2005). Valproate: A Simple Chemical with so Much to Offer. J. Clin. Pharm. Ther..

[20] Spina E., Perugi G. (2004). Antiepileptic Drugs: Indications Other than Epilepsy. Epileptic. Disord..

[21] Blaheta R.A., Michaelis M., Jr J.C., Herna P., Goethe-universita J.W., Goethe-universita J.W. (2005). Evolving Anticancer Drug Valproic Acid: Insights into the Mechanism and Clinical Studies. Med. Res. Rev..

[22] Moradzadeh M., Tabarraei A., Sadeghnia H. (2015). The Role of Histone Deacetylase (HDAC) as a Biomarker in Cancer. J. Mol. Biomark. Diagn..

[23] Seto E., Yoshida M. (2014). Erasers of Histone Acetylation: The Histone Deacetylase Enzymes. Cold Spring Harb. Perspect. Biol..

[24] Lee D.H., Ryu H., Won H., Kwon S.H. (2017). Advances in Epigenetic Glioblastoma Therapy. Oncotarget.

[25] Phiel C.J., Zhang F., Huang E.Y., Guenther M.G., Lazar M.A., Klein P.S. (2001). Histone Deacetylase Is a Direct Target of Valproic Acid, a Potent Anticonvulsant, Mood Stabilizer, and Teratogen*. J. Biol. Chem..

[26] Das C., Aguilera D., Vasquez H., Prasad P., Zhang M., Wolff J., Al E. (2007). Valproic Acid Induces P21 and Topoisomerase-II (α/β) Expression and Synergistically Enhances Etoposide Cytotoxicity in Human Glioblastoma Cell Lines. J. Neurooncol..

[27] Thotala D., Karvas R., Engelbach J., Garbow J., Hallahan A., DeWees T., Al E. (2015). Valproic Acid Enhances the Efficacy of Radiation Therapy by Protecting Normal Hippocampal Neurons and Sensitizing Malignant Glioblastoma Cells. Oncotarget.

[28] Tseng J.-H., Chen C.-Y., Chen P.-C., Hsiao S.-H., Fan C.-C., Liang Y.-C., Al E. (2017). Valproic Acid Inhibits Glioblastoma Multiforme Cell Growth via Paraoxonase 2 Expression. Oncotarget.

[29] Osuka S., Takano S., Watanabe S., Ishikawa E., Yamamoto T., Matsumura A. (2012). Valproic Acid Inhibits Angiogenesis In Vitro and Glioma Angiogenesis In Vivo in the Brain. Neurol. Med. Chir..

[30] Lee C.-Y., Lai H.-Y., Chiu A., Chan S.-H., Hsiao L.-P., Lee S.-T. (2016). The Effects of Antiepileptic Drugs on the Growth of Glioblastoma Cell Lines. J. Neurooncol..

[31] Garcia C.G., Romano I., Domith I., Silva D.C.L.E., Cossenza M., Kahn S.A., Geraldo L.H.M., dos Santos Assunção F., Romão L.F., Lima F.R.S. (2018). Combination Therapy with Sulfasalazine and Valproic Acid Promotes Human Glioblastoma Cell Death Through Imbalance of the Intracellular Oxidative Response. Mol. Neurobiol..

[32] Tam K.J., Hui D.H.F., Lee W.W., Dong M., Tombe T., Jiao I.Z.F., Khosravi S., Takeuchi A., Peacock J.W., Ivanova L. (2017). Semaphorin 3 C Drives Epithelial-to-Mesenchymal Transition, Invasiveness, and Stem-like Characteristics in Prostate Cells. Sci. Rep..

[33] Qi G., Lu G., Yu J., Zhao Y., Wang C., Zhang H., Xia Q. (2019). Up-Regulation of TIF1γ by Valproic Acid Inhibits the Epithelial Mesenchymal Transition in Prostate Carcinoma through TGF-β/Smad Signaling Pathway. Eur. J. Pharmacol..

[34] Karayan-Tapon L., Wager M., Guilhot J., Levillain P., Marquant C., Clarhaut J., Potiron V., Roche J. (2008). Semaphorin, Neuropilin and VEGF Expression in Glial Tumours: SEMA3G, a Prognostic Marker?. Br. J. Cancer.

[35] Peacock J.W., Takeuchi A., Hayashi N., Liu L., Tam K.J., Al Nakouzi N., Khazamipour N., Tombe T., Dejima T., Lee K.C. (2018). SEMA3C Drives Cancer Growth by Transactivating Multiple Receptor Tyrosine Kinases via Plexin B1. EMBO Mol. Med..

[36] Xu X., Zhao Z., Guo S., Li J., Liu S., You Y., Ni B., Wang H., Bie P. (2017). Increased semaphorin 3c expression promotes tumor growth and metastasis in pancreatic ductal adenocarcinoma by activating the ERK1/2 signaling pathway. Cancer Lett..

[37] Schindelin J., Arganda-Carrera I., Frise E., Verena K., Mark L., Tobias P., Stephan P., Curtis R., Stephan S., Benjamin S. (2012). Fiji—An Open Platform for Biological Image Analysis. Nat. Methods.

