# Supplementary material for: The Anti-Tumorigenic Activity of Sema3C in the Chick Embryo Chorioallantoic Membrane Model
Source: Int J Mol Sci. 2019 Nov 12;20(22):5672. doi: 10.3390/ijms20225672 (PMC6888630; doi:10.3390/ijms20225672)
Supplement: Supplementary file 1 [file ijms-20-05672-s001.pdf]

Supplementary Materials:

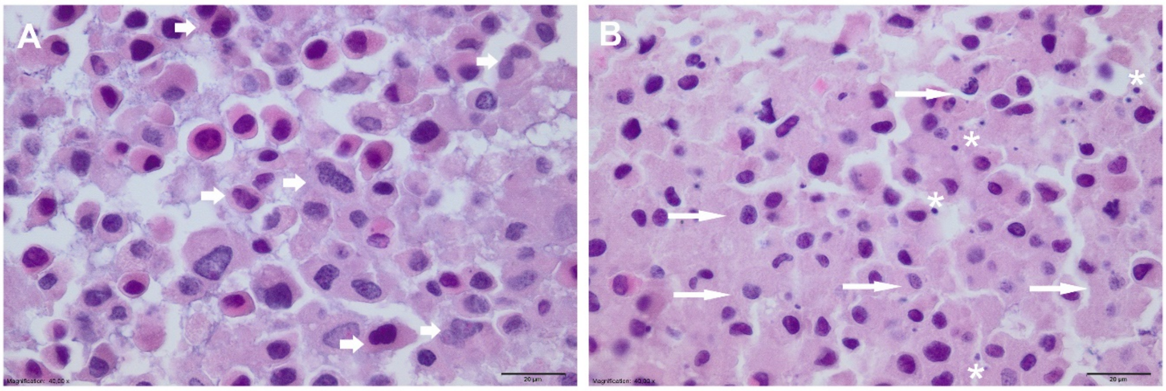

Supplementary figure S1. Microscopic images of the tumors on the CAM at x40 magnification. (**A**) CAM with U87t-Sema3C cell-formed tumor, (**B**) CAM with U87t-Sema3C tumor treated with 4 mM of NaVP. Non-treated tumor cells in (**A**) vary more in size, are bigger and more viable (mitotic cells are pointed with short arrows) than NaVP-treated cells in (**B**). NaVP treated cells in (**B**) are characterized by shrinkage, nuclear fragmentation (long arrows) and the appearance of apoptotic bodies (asterisks). Scale bar: 20 µm.

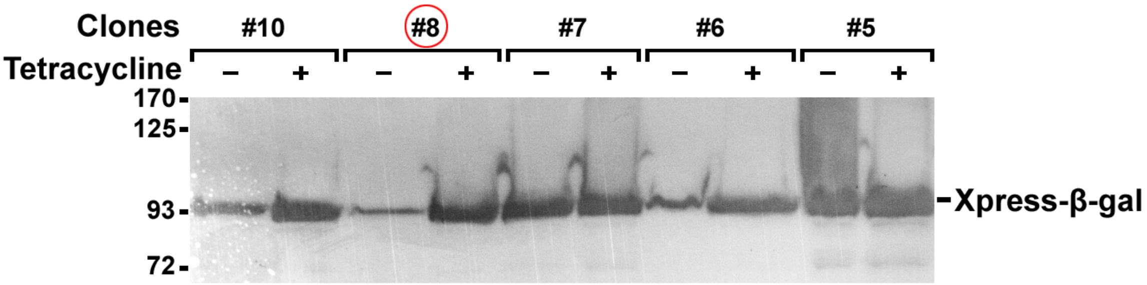

Supplementary figure S2. Western blot analysis of individual U87t clones. U87 MG cells were transfected with the TetR-encoding linearized pcDNA6/TR vector. After two-week selection with 6 µg/mL of blasticidin, individual U87t clones were selected and transfected with the vector pcDNA4/TO/lacZ encoding Xpress-tagged β-galactosidase driven by the tetracycline-regulated promoter. The transfected cells were treated with or without tetracycline for 48 h. Western blot analysis on whole-cell extracts prepared from transfected cells was carried out by using anti-Xpress antibodies. The tetracycline-dependent expression of Xpress-β-gal was most prominent in the clone #8 (marked with the red circle), which was chosen for further experiments under the name U87t. The protein band corresponding to Xpress-β-gal indicated on the membrane.
